# Correction: Inferring transmission heterogeneity using virus genealogies: Estimation and targeted prevention

**DOI:** 10.1371/journal.pcbi.1009359

**Published:** 2021-09-01

**Authors:** The PLOS Computational Biology Staff

## Notice of republication

This article was republished on October 8, 2020, to correct errors in equations and headings in the article PDF that were introduced during the typesetting process. The publisher apologizes for the errors. Please download this article again to view the correct version. The originally published, uncorrected article and the republished, corrected articles are provided here for reference.

## Supporting information

S1 File Originally published, uncorrected article. (PDF)

S2 File Republished, corrected article. (PDF)
