# Supplementary material for: Inferring transmission heterogeneity using virus genealogies: Estimation and targeted prevention
Source: PLoS Comput Biol. 2020 Sep 3;16(9):e1008122. doi: 10.1371/journal.pcbi.1008122 (PMC7494101; doi:10.1371/journal.pcbi.1008122)
Supplement: S1 Fig — Average branch length of the simulated tree under varied basic reproduction number (x-axis) and varied levels of heterogeneity (y-axis). Under each condition, the simulation started from one infection and stopped when there were 100 recovered individuals. The average branch length was calculated as the total branch length of the reconstructed tree over the number of tips. The results were the average over 1000 simulations. (PDF) [file pcbi.1008122.s001.pdf]

S1 Fig. Average branch length of the simulated tree under different conditions

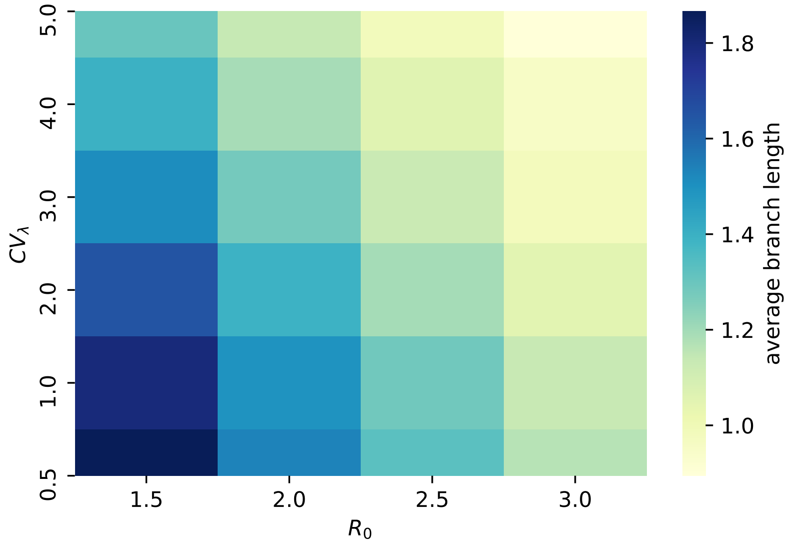

Average branch length of the simulated tree under varied basic reproduction number (x-axis) and varied levels of heterogeneity (y-axis). Under each condition, the simulation started from one infection and stopped when there were 100 recovered individuals. The average branch length was calculated as the total branch length of the reconstructed tree over the number of tips. The results were the average over 1000 simulations.
