# Supplementary material for: Inferring transmission heterogeneity using virus genealogies: Estimation and targeted prevention
Source: PLoS Comput Biol. 2020 Sep 3;16(9):e1008122. doi: 10.1371/journal.pcbi.1008122 (PMC7494101; doi:10.1371/journal.pcbi.1008122)
Supplement: S2 Fig — In each panel, the black line denotes the true value that was used to generate the simulated data. The colored curves are the means of the estimates under different levels of γ−1. The shaded area denotes 95% confidence interval estimated when γ−1 = 2.5. These results are obtained from 100 simulation replicates where the average transmissibility rate μλ was fixed as 1, the sequencing ratio ρ = 0.9, and the simulation stopped when there were 100 diagnosed individuals. The panel A is coefficient of variation (CVλ) and the panel B is standard deviation of infectivity rate (σλ). (PDF) [file pcbi.1008122.s002.pdf]

# S2 Fig. Inference of heterogeneity under various lengths of mean infectious period $\gamma^{-1}$

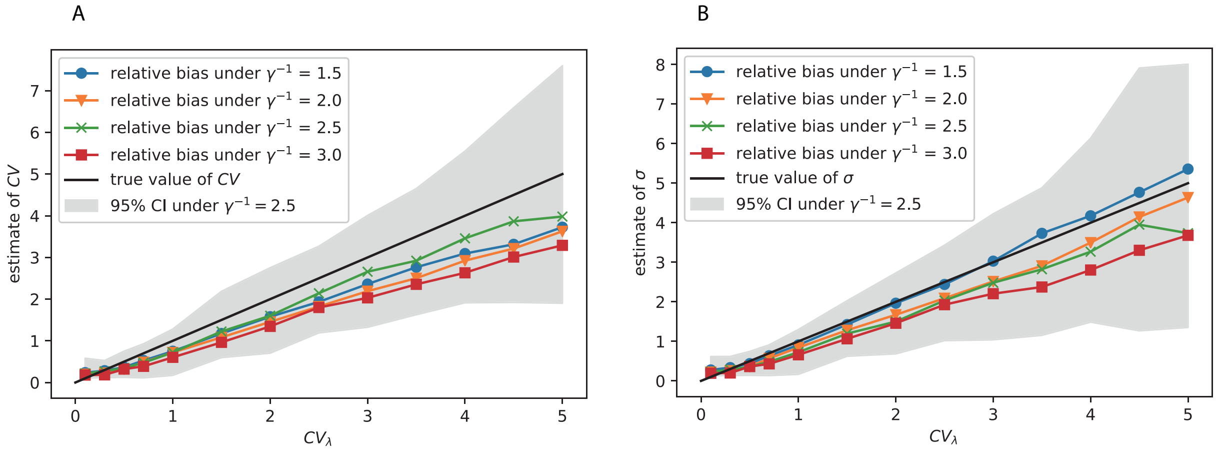


In each panel, the black line denotes the true value that was used to generate the simulated data. The colored curves are the means of the estimates under different levels of $\gamma^{-1}$. The shaded area denotes 95% confidence interval estimated when $\gamma^{-1} = 2.5$. These results are obtained from 100 simulation replicates where the average transmissibility rate $\mu_\lambda$ was fixed as 1, the sequencing ratio $\rho = 0.9$, and the simulation stopped when there were 100 diagnosed individuals. The panel A is coefficient of variation ($CV_\lambda$) and the panel B is standard deviation of infectivity rate ($\sigma_\lambda$).
