# Supplementary material for: Inferring transmission heterogeneity using virus genealogies: Estimation and targeted prevention
Source: PLoS Comput Biol. 2020 Sep 3;16(9):e1008122. doi: 10.1371/journal.pcbi.1008122 (PMC7494101; doi:10.1371/journal.pcbi.1008122)
Supplement: S3 Fig — In each panel, the colored curves are the means of relative biases (the bias over the true value) under different levels of γ−1. The shaded area denotes the relative bias of the 95% confidence interval estimated when γ−1 = 2.5. These results are obtained from 100 simulation replicates where the average transmissibility rate μλ was fixed as 1, the sequencing ratio ρ = 0.9, and the simulation stopped when there were 100 diagnosed individuals. A. basic reproduction number (R0), B. average infectivity rate (μλ), and C. recovery rate (γ). (PDF) [file pcbi.1008122.s003.pdf]

S3 Fig. Performance of parameter estimation under various lengths of mean infectious period γ^{-1}

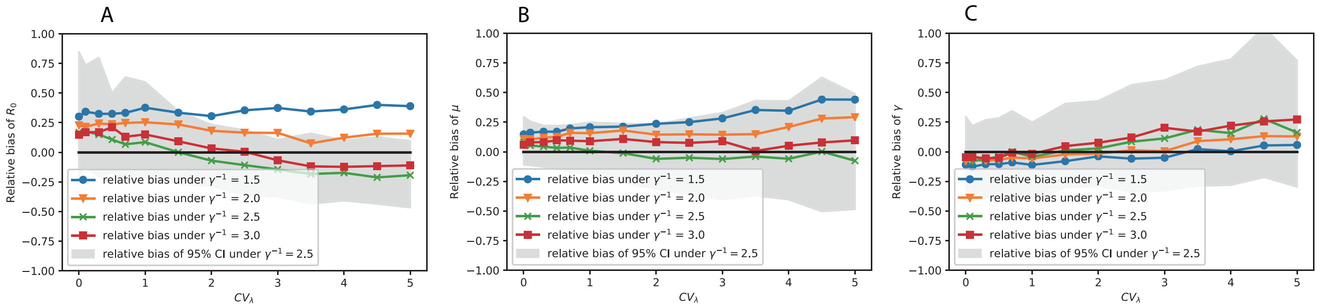

In each panel, the colored curves are the means of relative biases (the bias over the true value) under different levels of γ^{-1}. The shaded area denotes the relative bias of the 95% confidence interval estimated when $\gamma^{-1} = 2.5$. These results are obtained from 100 simulation replicates where the average transmissibility rate μ_λ was fixed as 1, the sequencing ratio $\rho = 0.9$, and the simulation stopped when there were 100 diagnosed individuals. A. basic reproduction number (R_0), B. average infectivity rate (μ_λ), and C. recovery rate (γ).
