# Supplementary material for: Inferring transmission heterogeneity using virus genealogies: Estimation and targeted prevention
Source: PLoS Comput Biol. 2020 Sep 3;16(9):e1008122. doi: 10.1371/journal.pcbi.1008122 (PMC7494101; doi:10.1371/journal.pcbi.1008122)
Supplement: S4 Fig — The lines with circles “○” are the estimate under the situation of constant transmissibility, and the shaded area denote the 95% confidence interval from 100 simulations. The lines with triangles “▽” are the estimates under the situation with time-varying transmissibility (TVT). The comparison is performed under the situation of R0 = 2.5 and the heterogeneity (CVλ) varies from 0 to 5. Sample size n = 100 and simulation runs = 100. (PDF) [file pcbi.1008122.s004.pdf]

S4 Fig. Comparison of parameter estimation under constant/time-varying transmissibility

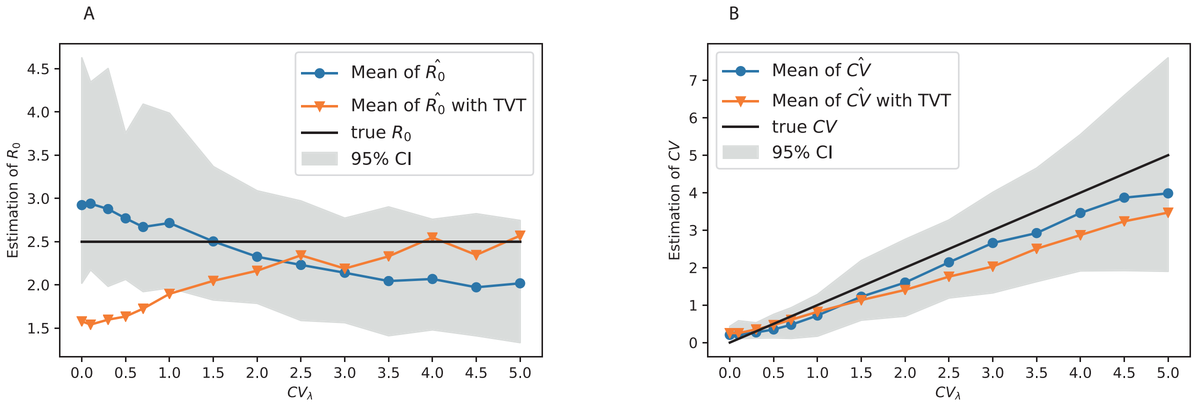

The lines with circles "○" are the estimate under the situation of constant transmissibility, and the shaded area denote the 95% confidence interval from 100 simulations. The lines with triangles "▽" are the estimates under the situation with time-varying transmissibility (TVT). The comparison is performed under the situation of $R_0 = 2.5$ and the heterogeneity (CV_λ) varies from 0 to 5. Sample size n= 100 and simulation runs = 100.
