# Supplementary material for: Inferring transmission heterogeneity using virus genealogies: Estimation and targeted prevention
Source: PLoS Comput Biol. 2020 Sep 3;16(9):e1008122. doi: 10.1371/journal.pcbi.1008122 (PMC7494101; doi:10.1371/journal.pcbi.1008122)
Supplement: S5 Fig — Four levels of threshold c have been calculated: m = 1 (blue), m = 2 (orange), m = 3 (green), and m = 4 (red). Results in (a) and (b) are the mean of 300 simulations. (PDF) [file pcbi.1008122.s005.pdf]

S5 Fig. Relative effect over random prevention of NCE_m strategy under the continuous monitoring scenario

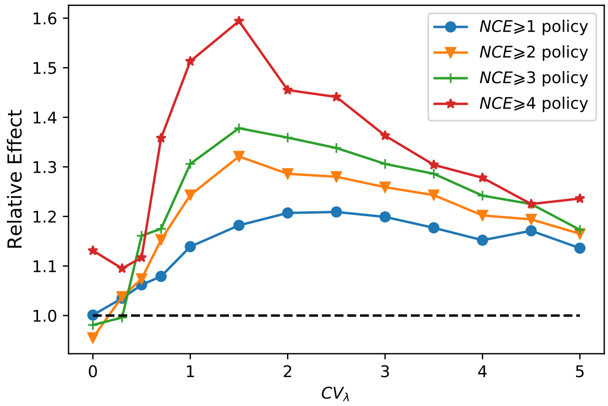

Four levels of threshold c have been calculated: $m = 1$ (blue), $m = 2$ (orange),$m = 3$ (green), and $m = 4$ (red). Results in (a) and (b) are the mean of 300 simulations.
