# Supplementary material for: Inferring transmission heterogeneity using virus genealogies: Estimation and targeted prevention
Source: PLoS Comput Biol. 2020 Sep 3;16(9):e1008122. doi: 10.1371/journal.pcbi.1008122 (PMC7494101; doi:10.1371/journal.pcbi.1008122)
Supplement: S6 Fig — Three levels of threshold m have been calculated: m = 1 (blue), m = 2 (orange), and m = 3 (green). Results are the mean of 300 simulations. (PDF) [file pcbi.1008122.s006.pdf]

S6 Fig. Comparison of fraction of contact traced of NCE_m strategy under the situation of with/without within-host diversity (solid/dash lines respectively).

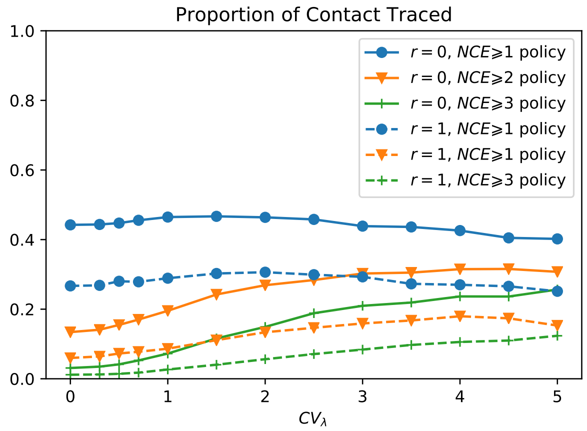

Three levels of threshold m have been calculated: $m = 1$ (blue), $m = 2$ (orange), and $m = 3$ (green). Results are the mean of 300 simulations.
