# Supplementary material for: Inferring transmission heterogeneity using virus genealogies: Estimation and targeted prevention
Source: PLoS Comput Biol. 2020 Sep 3;16(9):e1008122. doi: 10.1371/journal.pcbi.1008122 (PMC7494101; doi:10.1371/journal.pcbi.1008122)
Supplement: S8 Fig — Computing times (y-axis) required for processing trees with varying numbers of tips (each tip represents a diagnosed individual). For each number of tips (x-axis), we simulated trees with different levels of heterogeneity (i.e., CVλ = 1, 2, ⋯, 5). Each point represents the average computing time based on 10 simulations with given heterogeneity level and the size of tree. The number of tips was varied along the following sequence: 100, 200, 400, 1000, 2000, 4000. The dashed line represents the average computing time over all levels of heterogeneity. All runs were executed on an Intel Core i7 processor (Mac mini 2018). (PDF) [file pcbi.1008122.s008.pdf]

S8 Fig. Computing times required for inferring heterogeneity from trees with varying numbers of tips.

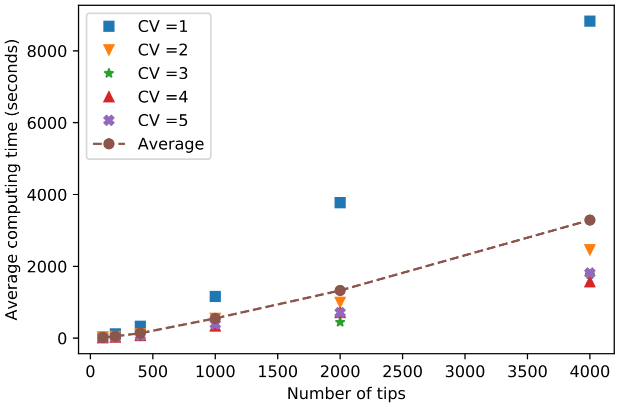

Computing times (y-axis) required for processing trees with varying numbers of tips (each tip represents a diagnosed individual). For each number of tips (x-axis), we simulated trees with different levels of heterogeneity (i.e., $CV_\lambda = 1, 2, \cdots, 5$). Each point represents the average computing time based on 10 simulations with given heterogeneity level and the size of tree. The number of tips was varied along the following sequence: 100, 200, 400, 1000, 2000, 4000. The dashed line represents the average computing time over all levels of heterogeneity. All runs were executed on an Intel Core i7 processor (Mac mini 2018).
