# Supplementary material for: Inferring transmission heterogeneity using virus genealogies: Estimation and targeted prevention
Source: PLoS Comput Biol. 2020 Sep 3;16(9):e1008122. doi: 10.1371/journal.pcbi.1008122 (PMC7494101; doi:10.1371/journal.pcbi.1008122)
Supplement: S9 Fig — Virus genealogies were simulated under two scenarios: without autocorrelation in transmission rates (A and B) and with autocorrelation (C and D). In the former cases, each infected individual independently draws its transmission rate from a binomial distribution, i.e., choosing the slow rate λ1 with probability 1 − πc = 0.9 and choosing the fast rate λ2 with probability πc = 0.1. In (A), λ1 = 2 and λ2 = 6, corresponding to mean rate of μλ = 2.4 and a low level of heterogeneity (CVλ = 0.5). In (B)λ1 = 1 and λ2 = 15, corresponding to mean rate of μλ = 2.4 and a high level of heterogeneity (CVλ = 1.75). In the cases with autocorrelation in transmission rates, each newly infected individual switch its transmission rate with probability πs and remains its infectee’s rate with probability 1 − πs. In both (C) and (D), λ1 = 0.9 and λ2 = 8.1 with different switching probability, i.e., πs = 0.2 in (C), corresponding to μλ = 5.22 and low level heterogeneity CVλ = 0.79, and πs = 0.8 in (D) corresponding to μλ = 2.64 and low level heterogeneity CVλ = 1.27. And the diagnosis rate is fixed as γ = 1. The x- and y-axes correspond to the estimated μλ and the estimated CVλ respectively. Each point represents the outcome when analyzing one of 100 replicates. (PDF) [file pcbi.1008122.s009.pdf]

**S9 Fig. Performance of new method and the Markov-modulated Poisson process (MMPP) based genetic clustering method on simulated data.**

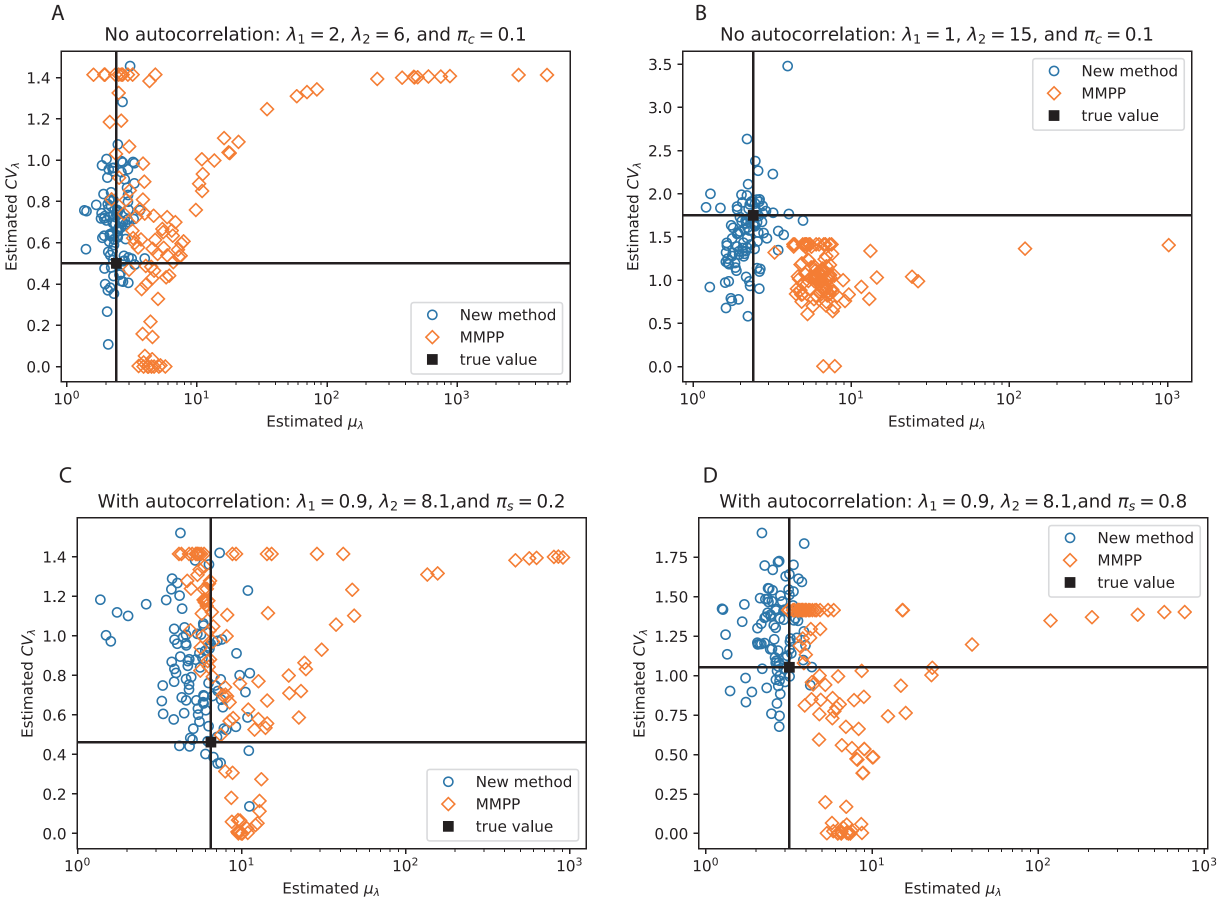


Virus genealogies were simulated under two scenarios: without autocorrelation in transmission rates (A and B) and with autocorrelation (C and D). In the former cases, each infected individual independently draws its transmission rate from a binomial distribution, i.e., choosing the slow rate $\lambda_1$ with probability $1 - \pi_c = 0.9$ and choosing the fast rate $\lambda_2$ with probability $\pi_c = 0.1$. In (A), $\lambda_1$=2 and $\lambda_2 = 6$, corresponding to mean rate of $\mu_\lambda = 2.4$ and a low level of heterogeneity ($CV_\lambda = 0.5$). In (B)$\lambda_1 = 1$ and $\lambda_2 = 15$, corresponding to mean rate of $\mu_\lambda = 2.4$ and a high level of heterogeneity ($CV_\lambda = 1.75$). In the cases with autocorrelation in transmission rates, each newly infected individual switch its transmission rate with probability $\pi_s$ and remains its infectee's rate with probability $1 - \pi_s$. In both (C) and (D), $\lambda_1 = 0.9$ and $\lambda_2 = 8.1$ with different switching probability, i.e., $\pi_s = 0.2$ in (C), corresponding to $\mu_\lambda = 5.22$ and low level heterogeneity $CV_\lambda = 0.79$, and $\pi_s = 0.8$ in (D) corresponding to $\mu_\lambda = 2.64$ and low level heterogeneity $CV_\lambda = 1.27$. And the diagnosis rate is fixed as $\gamma = 1$. The x- and y-axes correspond to the estimated $\mu_\lambda$ and the estimated $CV_\lambda$ respectively. Each point represents the outcome when analyzing one of 100 replicates.
