# Supplementary material for: Inferring transmission heterogeneity using virus genealogies: Estimation and targeted prevention
Source: PLoS Comput Biol. 2020 Sep 3;16(9):e1008122. doi: 10.1371/journal.pcbi.1008122 (PMC7494101; doi:10.1371/journal.pcbi.1008122)
Supplement: S1 Text — Includes details on simulating the virus genealogy, how the correction for incomplete transmission chain were derived, the preprocess of the real epidemiological data from Sweden, how the evaluation of control measures was performed based on simulation, and the pseudo-code of the proposed algorithm. (PDF) [file pcbi.1008122.s010.pdf]

Supplementary Information
Inferring transmission heterogeneity using virus genealogies: estimation and targeted prevention

Yunjun Zhang[a,b], Thomas Leitner[c], Jan Albert[d,e], and Tom Britton[b]

[a] *Department of Biostatistics, School of Public Health, Peking University, Beijing, China*
[b] *Department of Mathematics, Stockholm University, Stockholm, Sweden*
[c] *Theoretical Biology and Biophysics Group, Los Alamos National Laboratory, Los Alamos, New Mexico, USA*
[d] *Department of Microbiology, Tumor and Cell Biology, Karolinska Institute, Stockholm, Sweden*
[e] *Department of Clinical Microbiology, Karolinska University Hospital, Stockholm, Sweden*

August 23, 2020

S1 Simulating Virus Genealogy

S1.1 Overview of Simulating Genealogy

Given a set of model parameters (i.e., γ, μ_λ, and σ_λ), we began the simulation with one infection at time $t = 0$. For the i-th infected individual, we draw a random transmissibility rate $\lambda_i \sim \Gamma(\mu_\lambda, \sigma_\lambda)$ and a random infectious period $L_i^I \sim Exp(\gamma)$. The infections caused by the i-th infected individual within its infectious period follow a *Poisson* process with rate λ_i. Here the infectious period is referred to as the period from the time of infection to that of diagnosis. We used Gillespie's next-reaction method [3, 5] to simulate all these transmission events and diagnosis events from which a transmission history $\mathcal{H}$ is obtained.

If there is no within-host diversity, a complete virus genealogy $\mathcal{G}$ is identical to the transmission tree $\mathcal{T}$ which corresponds to the transmission history $\mathcal{H}$ without direction of transmission information. Thus, the complete virus genealogy $\mathcal{G}$ is generated by omitting the direction of transmission in $\mathcal{H}$. Furthermore, we randomly sample a fraction ρ_{SD} of the diagnosed cases in the complete genealogy to

generate a sampled virus genealogy $\mathcal{G}^{(S)}$ by suppressing all edges without sampled descendants [4].

The procedures of simulating a genealogy in the presence of within-host diversity or under the situation of finite susceptible individuals are similar, see the following description.

S1.2 Simulating a virus genealogy in the presence of within-host diversity

In the presence of within-host diversity, the transmission tree $\mathcal{T}$ and the complete viral genealogy $\mathcal{G}$ are not identical. We instead used the same method as in [2] to generate the virus genealogy $\mathcal{G}$ in the presence of within-host variation. This simulation uses a transmission history $\mathcal{H}$ as input, and generates the complete virus genealogy $\mathcal{G}$ according to a two-phase coalescent model described by a linear growth from a single transmitted variant (transmission bottleneck) to a maximum population size, followed by either stabilisation or decline of the effective population size. As for the situation of no within-host diversity, there are two types of individuals in the complete genealogy $\mathcal{G}$: one is the diagnosed and non-infectious, the other is undiagnosed and still infectious. We then randomly selected a sample of diagnosed cases with the sequencing ratio of ρ_{SD} to reconstruct the sampled genealogy $\mathcal{G}^{(S)}$. Specifically, the sampled genealogy $\mathcal{G}^{(S)}$ is then generated by suppressing all edges in $\mathcal{G}$ without sampled descendants as in [4].

S1.3 Simulating a virus genealogy under finite population size

We update the procedure of simulating a transmission history to adapt to a finite susceptible population size. Under this condition, infectious individuals have "infectious contact" with other individuals randomly in time at their own rate, and each such contact is with a randomly selected individual. That is, if the contacted individual is susceptible at the time of being contacted, then a new infection is generated. Therefore, the simulation has been performed in three layers: firstly, we simulate the occurrence of infectious contacts from infectious individuals with the heterogeneous birth-death model; secondly, when there is an infectious contact, we draw a uniform random number u. If u is smaller than the proportion of susceptible individuals in the whole population at that time, which means that a susceptible individual is contacted, then a transmission event is generated; otherwise the contact has no effect and the simulation continues until the occurrence of next infectious contact. Finally, based on the simulated transmission history under the finite population size, a sampled genealogy can be generated as before.

S2 Correction for Incomplete Transmission Chain

In this section, we derive the method of correction for incomplete transmission chain in equation (6)-(8) in the main manuscript. Before correction, we applied the Coordinate-ascent algorithm to the local genealogy $\mathcal{G}^{(S)}_{t-L_p}$ (the local part of $\mathcal{G}^{(S)}$ up to time $t - L_p$) by assuming all individuals infected within $[0, t - L_p]$ are sampled. The outcome estimates were denoted as μ^0_λ and σ^0_λ. In addition, we estimated the sampling ratio of $\mathcal{G}^{(S)}_{t-L_p}$ to $\rho = \rho_{SD} * p$, that is, there was a fraction $1-\rho$ of the infected individual which were not sampled in $\mathcal{G}^{(S)}_{t-L_p}$. We now introduce a correction of μ^0_λ and σ^0_λ by allowing for these unsampled individuals.

We let n denote the number of tips in $\mathcal{G}^{(S)}_{t-L_p}$, with the raw estimates of transmission rates as $\{\hat{\lambda}_i\}^n_{i=1}$. The expected number of unsampled individuals equals $m = n * (1/\rho - 1)$.

This correction has been performed on the basis of data imputation. For the unsampled individuals (including the undiagnosed individuals and the diagnosed but unsequenced individuals), it is possible to set their transmissibility rates to a constant value (denoted as λ_{us}), that is, the corrected transmissibility rates for the unsampled individuals are $\hat{\lambda}^p_{n+j} = \lambda_{us}$ $(j = 1, \cdots, m$. The value of λ_{us} has already been discussed in the main text. On the other hand, for the sampled individuals, their estimated transmissibility rates shall be upscaled if allowing for partial sampling, that is, the corrected transmissibility rates (denoted $\hat{\lambda}^p_i$) for the sampled individuals are $\hat{\lambda}^p_i = \hat{\lambda}_i/\rho$ $(i = 1, \cdots, n)$.

Based on these assumptions, the corrected estimate of average transmissibility rate (denoted as $\hat{\mu}^p_\lambda$) will be calculated as follows:

$$\hat{\mu}^p_\lambda = \frac{\sum^n_{i=1} \hat{\lambda}^p_i + \sum^m_{j=1} \hat{\lambda}^p_{n+j}}{n+m} = \frac{n\hat{\mu}_\lambda/\rho + m\lambda_{us}}{n+m} = \hat{\mu}_\lambda + (1-\rho)\lambda_{us}$$

In addition, the corrected estimate of the heterogeneity, measured by the standard deviation of the infectivity rate, is:

$$\begin{aligned}
\hat{\sigma}^p_\lambda &= \sqrt{\frac{\sum^n_{i=1}(\hat{\lambda}^p_i - \hat{\mu}^p_\lambda)^2 + \sum^m_{j=1}(\hat{\lambda}^p_{n+j} - \hat{\mu}^p_\lambda)^2}{n+m}} \\
&= \sqrt{\frac{\sum^n_{i=1}(\hat{\lambda}_i/\rho - \hat{\mu}_\lambda - (1-\rho)\lambda_{us})^2 + \sum^m_{j=1}(\lambda_{us} - \hat{\mu}_\lambda - (1-\rho)\lambda_{us})^2}{n+m}} \\
&= \sqrt{\frac{\hat{\sigma}^2_\lambda}{\rho} + \frac{1-\rho}{\rho}(\hat{\mu}_\lambda - \rho\lambda_{us})^2}
\end{aligned}$$

where λ_{us} is the substituted transmissibility rate for the unsampled individuals. And we set that λ_{us} varies with the level of heterogeneity as

$$\lambda_{us} = \frac{\mu_\lambda}{\rho} + \frac{1 - \exp(\widehat{CV}^0_\lambda)}{1 + \exp(\widehat{CV}^0_\lambda)}(\hat{\gamma} - \frac{\mu_\lambda}{\rho}),$$

where $\widehat{CV}^0_\lambda = \hat{\sigma}^0_\lambda / \mu^0_\lambda$ is a raw estimate. In addition, the derivation of the equations (7)-(9) in the manuscript is obtained by replacing the sampling ratio ρ with $\rho_{SD} * p$.

Finally, the corrected estimate of the Coefficient of Variation is

$$\widehat{CV}^p_\lambda = \frac{\hat{\sigma}^p_\lambda}{\hat{\mu}^p_\lambda}$$

S3 Real epidemiological data and genealogical reconstruction

Three HIV-1 sequence sets (IDU_AE, IDU_B and MSM_B) were analyzed in this study. As in [2], the time-scaled virus phylogenies were generated using a Bayesian Skyline coalescent model in BEAST 1.8 using the general time reversible nucleotide substitution model with an uncorrelated log-normal relaxed clock and a discretized gamma distribution with four categories. For the log-normal relaxed clock parameters, a uniform prior on the positive axis was assumed for the mean, and an exponential with mean 1/3 for the standard deviation. A Uniform prior on (0, 1) was used for the nucleotide frequencies. The Markov Chain Monte Carlo algorithm was run for 10 million iterations, with a 10% burn-in period and samples saved every 10000 iterations. For each data set, a random sample of 10000 genealogies were drawn from the posterior distribution of genealogies. Particularly, for the IDU_B dataset, we assumed that the estimated average time to diagnosis was shorter than 20 years, so there were 7493 genealogies which were used in the inference.

S4 Simulation Study of Epidemic Control

In the simulation study of epidemic control, we first simulated an epidemic beginning with one infection and stopped at the time when there were 100 individuals had been diagnosed (the time was denoted as t_I). During this period, there was no prevention.

We then continued the simulation for another L_{cp} time units under certain prevention. The second period of simulation is referred to as *control period.* In

our study, the control period equals $L_{cp} = 2.5$ years (this being the average time to diagnosis for HIV-1 in Sweden).

S4.1 Evaluation of Prevention Strategies

We considered two situations of epidemic control: one is the 'continuous monitoring' as the typical public health situation where samples (virus sequences from diagnosed cases) are continuously collected throughout an epidemic, and where for each collected sample between t_I and $t_I + L_{cp}$ it is decided to perform (additional) contact tracing or not. The second situation is denoted 'cross-sectional sampling' which is the situation where all samples are collected at time t_I when 100 individuals had been diagnosed. Among the sampled individuals at time t_I, we selected a fraction of them for contact tracing.

Under the situation of continuous monitoring sampling, the phylogeny-guided prevention was compared with the random prevention and no prevention. When considering the cross-sectional sampling, another prevention strategy denoted MRD, selecting the Most Recently Diagnosed individuals, was also simulated for comparison.

For a particular prevention strategy, its effectiveness at time τ ($0 < \tau < L_{cp}$) (where L_{cp} is the length of the control period) is defined as the *relative epidemic size* (RES), that is,

$$RES_{\tau} = \frac{\#(\text{new infections by } \tau | \text{prevention strategy})}{\#(\text{new infections by } L_{cp} | \text{no prevention})} \tag{1}$$

where #(new infections by τ|prevention strategy) is the number of infected individuals during the period of $[0,\ \tau]$ under the particular prevention strategy. Furthermore, the relative effect of a particular contact tracing strategy over the random contact tracing is defined as

$$\text{Relative effect} = \frac{1 - RES_{L_{cp}}(\text{prevention strategy})}{1 - RES_{L_{cp}}(\text{random prevention})}.$$

We calibrated all these prevention strategies to have the same *fraction of contact traced*, defined as the fraction of diagnosed individuals being selected for contact tracing of recent contacts. The details of calibration will be described in the next section.

S4.2 Calibration of Prevention Strategies

To make a fair comparison, we do calibrate the different strategies by having the same *number* of individuals for contact tracing, but different particular individualsfor the different strategies. An individual being selected for contact tracing

is referred to as an index case, and the ratio of the number of index cases to the total number of (sampled/sequenced) individuals is referred to as the *fraction of contact traced* (denoted as ρ_{ct}).

Under the cross-sectional situation, contact tracing is only performed at the beginning of the control period (i.e., at time t_I). Hence the calibration is only performed at time t_I. Among all the sampled individuals up to time t_I, we select a fraction of ρ_{ct} for contact tracing either randomly (random prevention), or based on the descending order of NCE value (NCE based prevention), or based on the descending order of times since diagnosis (MRD prevention). Recall that NCE method counts the number of coalescent events within $t_c = 1.25$ years prior to being diagnosed, and selects individuals having m or more such events (where m is either 1, 2 or 3).

Under the situation of continuous monitoring, the contact tracing is implemented continuously in time, hence it is impossible to estimate how many sampled individuals will be selected for contact tracing under the NCE based prevention. Therefore, the calibration consists of two steps. Firstly, we perform the simulation under the NCE policy which starts at time t_I and lasts for L_{cp} time length. During this simulation, when there is a newly sampled individual, the corresponding NCE value is calculated and the decision of contact tracing has been made based on the NCE value. Based on this simulation, we estimate the corresponding fraction of contact traced ρ_{ct} which will be used to calibrate the simulation under the random contact tracing.

Secondly, we perform the simulation under the random contact tracing strategy which starts at time t_I as before and lasts also for L_{cp} time length. During this simulation, when there is a newly sampled/sequenced individual, it will be selected for contact tracing randomly with the probability ρ_{ct} estimated at the first step. This way the random policy and NCE policy will have the same fraction of contact traced under the situation of continuous monitoring.

S4.3 Scheme of prevention

The default scheme of prevention is *single-step* contact tracing, that is, the direct contacts of the index case within a fixed period (here the period is equal to t_c) will, in the case they have been infected, be diagnosed and isolated to stop infection. The potential individuals to be prevented include the secondary infections caused by the index case as well as the individual who infected the index case if it was infected within the period of t_c since diagnosis. The single-step contact tracing might be applicable when there is no simple and rapid diagnostic test available [1]. A more common situation is however *iterative* contact tracing. This is when those individuals who are initially contact traced that are found infected are treated as new index cases, i.e. where contacts of these individuals are also sought, and so

on [1]. We also made a comparison between these two schemes of contact tracing to show the full beneficial effects of phylogeny-guided prevention. Note that the fraction of contact traced for the iterative contact tracing is calculated based on the original index cases, implying that more individuals are contact traced in the iterative case as compared to the single-step case.

S5 Pseudo-code

S5.1 Pseudo-code of the Coordinate Ascent algorithm

Data: A complete genealogy $\mathcal{G}$ with n tips
Result: The estimated parameters $\Theta = (k, \theta, \gamma)$
Calculating the heights of all branches in $\mathcal{G}$, and denoting the branches with the height of j as the j-th slice;
Calculating the partial transmission history of n individuals as $\hat{\mathcal{H}}^0 = \{(x_i^0, d_i^0)\}_{i=1}^n$ (where $x_i^0 = 0$) by assigning the external branches (i.e., the $j = 0$ slice) to different individuals
Calculating initial estimate $\hat{\Theta}^0$ according to eq (2) and (3) based on $\hat{\mathcal{H}}^0$.
for $i = 1, \cdots, s$ **do**
  while *the estimate of* $\hat{\Theta}^m$ *does not converges* **do**
    for *each branch in* j*-th slice* **do**
      evaluating its labelling coefficient as eq (4) based on $\hat{\Theta}^m$ and $\hat{\mathcal{H}}^m$
    end
    Updating the reconstructed transmission history $\hat{\mathcal{H}}^m$ according to the labelling coefficients;
    Updating the estimate of $\hat{\Theta}^m$ according to eq (2) and (3) based on the updated $\hat{\mathcal{H}}^m$;
  end
end
Deliver the final estimate $\hat{\Theta} = \hat{\Theta}^s$

S5.2 Pseudo-code of Analysis of Sampled Genealogy

Data: A sampled genealogy $\mathcal{G}^{(s)}$ and a serial of censoring probability $\{p_i\}_{i=1}^{M}$
Result: The corrected estimation $\hat{\Theta}^c$
for $m = 1, \cdots, M$ **do**
    Calculating L_{p_i} as the p_i percentile for the lengths of external branches in $\mathcal{G}^{(s)}$;
    Obtaining the local genealogy $\mathcal{G}^{(s)}_{t-L_{p_i}}$ as the part of $\mathcal{G}^{(s)}$ up to time $t - L_{p_i}$
    Calculating the raw estimate $\hat{\Theta}^0_i = (\hat{\mu}^0_\lambda, \hat{\sigma}^0_\lambda, \hat{CV}^0)$ by applying the coordinate algorithm to $\mathcal{G}^{(s)}_{t-L_{p_i}}$ without considering the unsampled individuals
    Calculating the corrected estimate $\hat{\Theta}^p_i = (\hat{\mu}^p_\lambda, \hat{\sigma}^p_\lambda, \hat{CV}^p)$ as eq (6)-(9) based on $\hat{\Theta}^0_i$ and the corresponding sampling ratio $p_i * \rho_{SD}$
end
Calculating the average estimate as $\hat{\Theta}^c = \frac{\sum_{i=1}^{M} \hat{\Theta}^p_i}{M}$

References

[1] Eames, K. T. D. Contact tracing strategies in heterogeneous populations. *Epidemiology and Infection 135*, 3 (2007), 443–454.

[2] Giardina, F., Romero-Severson, E. O., Albert, J., Britton, T., and Leitner, T. K. Inference of transmission network structure from hiv phylogenetic trees. *PLOS Computational Biology 13*, 1 (2017), 59865.

[3] Gillespie, D. T. Exact stochastic simulation of coupled chemical reactions. *The journal of physical chemistry 81*, 25 (1977), 2340–2361.

[4] Stadler, T., Kouyos, R., von Wyl, V., Yerly, S., Böni, J., Bürgisser, P., Klimkait, T., Joos, B., Rieder, P., Xie, D., et al. Estimating the basic reproductive number from viral sequence data. *Molecular biology and evolution 29*, 1 (2011), 347–357.

[5] Wilkinson, D. J. *Stochastic modelling for systems biology.* Chapman and Hall/CRC, 2006.
