# Supplementary material for: Inferring transmission heterogeneity using virus genealogies: Estimation and targeted prevention
Source: PLoS Comput Biol. 2020 Sep 3;16(9):e1008122. doi: 10.1371/journal.pcbi.1008122 (PMC7494101; doi:10.1371/journal.pcbi.1008122)
Supplement: Attachment — Submitted filename: Re to Reviewer0204.docx [file pcbi.1008122.s011.docx]

*We thank the reviewers for careful reading and giving us many suggestions for improvement! We have modified the text accordingly, and also corrected a few other minor things. Below we outline how the text has been modified based on each point raised by the referees. Our comments are written in italics.*

**Reviewer #1**: This manuscript describes a new method to detect variation in transmission rates from the shape of the phylogenetic tree reconstructed from pathogen sequences. This quantity has public health relevance because it may enable one to optimize the allocation of prevention resources by focusing on those individuals with the highest probability of onward transmission, based on past dynamics.

More specifically, the "heterogeneous birth-death" model presented by the authors is an extension of the standard birth-death model to accommodate variation in transmission rates among individuals through a random-effects approach, where these rates are drawn from a gamma distribution to be parameterized from the data. This random-effects approach is distinct from that of otherwise similar models, such as Kuhnert’s two-type birth-death model (PLOS Pathog 14(2): e1006895), Barido-Sottani’s multi-state birth-death model (J R Soc Interface 15(146)) or McCloskey’s pure-birth model (PLOS Comput Biol 13(11): e1005868), where lineages “evolve” between latent discrete branching rate states. Notably, none of these publications are cited by the authors. (See also a preprint from Volz et al.; bioRxiv, https://doi.org/10.1101/704528). Since the authors are presenting a new method, they should also evaluate their method alongside at least one of these other approaches that are generally designed to accomplish the same objective.

***Re:*** *Thanks for this suggestion! We gave a review on these interesting works in the “Introduction” (Page 2 line 19--34) and made a comparison on simulated data with one of these method in the section of “Result”(Page 10 line 364--384).*

Furthermore, the rates in the heterogeneous model are distributed among individuals in an uncorrelated fashion with respect to the transmission history, whereas the rates are autocorrelated in the two-type and multi-state models. This difference highlights the focus of this manuscript on the individual host, rather than groups in a risk-structured population. The assumption of uncorrelated transmission rates (absence of homophily by risk) needs to be justified (e.g., is it appropriate for HIV-1), as it is a distinctive feature of this model. The authors should also evaluate the impact of variation in diagnosis rates among individuals (rate parameter $\gamma$) on their power to detect heterogeneity in transmission rates (more below).

***Re:*** *Yes, it is possible to consider correlation in transmissibility between infector and infectee. Our model does not consider this correlation because our model was motivated by the variation in virus loads among individuals. We added this description in the section of “Introduction”(Page 3 line 45 -- 52).*

An important starting assumption of the model is that transmission unfolds in an infinite population of susceptible hosts, which supports the approximation that “transmission events occur independently” (line 164), for instance. The authors evaluate their model when this assumption is relaxed - however, this was carried out in the initial exponential growth phase of the epidemic (starting from a single infected individual to an average of 120 infections in a total population of 1000 hosts). This minimizes the effect of limited susceptibles, i.e., the “collision” of infected lineages, and it would be helpful to see the robustness of the model when the epidemic is at a more intermediate stage.

***Re:*** *In the simulation with finite population size, we started from a single infected individual and stopped when there were on average 220 infected individuals (100 diagnosed and 120 undiagnosed). So the average prevalence is about 22% (about 220 infected individuals over 1000 initial susceptible individuals) which quite high. (Page 10 line 398--400).*


Another, more problematic assumption of this model is that the expected time to diagnosis is the same across all individuals. Incomplete and inevitably biased sampling (diagnosis) is widely recognized as a severe confounding problem in measuring variation in transmission rates. For example, if individuals with higher transmission rates are also more likely to be lost to follow-up for sampling (sequencing after diagnosis), then the model (and most other models as well) will suffer from a systematic loss of sensitivity. This limitation should be addressed in the Discussion section.

***Re:*** *Thanks for this suggestion! We agree that diagnoses occur at different times after infection for different persons. We have now made that clearer and discuss this in the discussion (Page 17 line 697--707).*

While the authors evaluated the assumption of incomplete sampling by allowing for transmission to unsampled individuals, they also assume that there is no onward transmission from unsampled to sampled individuals - viz., from the Supplementary Text S1: “Furthermore, we assume that the unsampled individuals have small level of transmissibility rates and that they caused no infection before being diagnosed.” This is an unrealistic assumption and especially problematic in the case of targeting specific individuals for public health intervention – to say nothing of the potential exposure of individuals to stigma and criminalization – and it should be described more transparently in the manuscript (for instance, around lines 221-223) and rationale given.

***Re:*** *We thank the referee for pointing this out. In the later part of the subsection “Analysis of sampled genealogy” in the manuscript (Page 8 line 261-280), we provided a more detailed explanation about how we assume the transmission rates for the unsampled individuals.*

The GitHub repository should incorporate the documentation in the Word document (which is a binary file) into the README in the conventional Markdown format. In addition, I found that the Python scripts do not adhere to PEP8 (e.g., lack of whitespace separating arguments) and there is a general lack of inline or block comments that would make it easier to review the code. Variables such as “mu_RecEst” need to be defined within the source code. There are multiple uses of undocumented magic numbers (e.g., 9999) where it is more conventional to use the Python Nonetype or math.inf. The module documentation needs to provide installation instructions, including module dependencies such as matplotlib and scipy. The tutorial is incomplete and should provide instructions for loading the module prior to calling functions such as “singleSimu”.

***Re:*** *Thanks for these suggestions! We revised the codes in the GitHub repository, including rewriting the READEME file, adding more comments to make it easier to read, and using math.inf to define the extreme situation. Please go to the GitHub （*github.com/yunPKU/HeteroInfer *） for details.*

In my small experiments with these scripts, I found that mean $\lambda$ was substantially underestimated when setting $\mu$ to 3.0 - the average estimate of 5 trials was 1.57 ($\gamma$ set to 0.3). This may be masked by reporting accuracy for CV and not the mean, particularly when the variation parameter is underestimated the same way. Please provide results for these parameters individually. There also seems to be some interaction with $\gamma$; if I set this parameter to 0.7 then the mean estimate of $\mu$ increased to 1.99. This effect on parameter identifiability should be explored.

***Re:*** *Thanks for this suggestion. We provided more simulation results under several levels of R0 in the “Results” (Page 9 line 352—363).*

I could not find details on run times of the analysis or estimates of time complexity. It took roughly 10 seconds to simulate and analyze a single tree with sample size 100 (default setting) on a rather outdated iMac (late 2013), and about 1 minute for a sample of 200, so I suspect that the complexity will be quadratic or more with sample size. An assessment of computing time should be incorporated into the revision.

***Re:*** *Thanks for your suggestion! We added the analysis of computing time in the subsection “Computing time” (Page 12 line 486-502).*

As developers and users of phylogenetic methods in the context of HIV-1, we are obligated to discuss the ethical implications of work that can be potentially used for source attribution (i.e., “who infected whom”). Specifically, this study proposes to identify individuals who are associated with higher numbers of coalescent events, which can be loosely interpreted as transmission events, as targets for public health intervention. In light of ongoing criminalization of HIV in many countries worldwide, and where punishments are often disproportionately severe, it is inevitable that methods such as the one presented by the authors here will also be used in the prosecution of individuals for transmission without disclosure. The authors should address this problem in the discussion section of their revised manuscript.

***Re:*** *Thanks for this suggestion! We are aware of the ethical dilemma scientists in this, and many other, fields have, developing better methods for inference when such methodology may be abused. We have added a comment on this in the discussion (line Page 16 line 690--693).*

Overall, I found this an interesting manuscript. It is written well and I found the model description mostly straight-forward. As discussed above, there are a number of potentially (or almost certainly) problematic model assumptions that need to be explored further, and the authors should address other work in this area and perform some comparison of methods.

- line 64: Is there any empirical motivation for using the gamma distribution to model variation in transmission rates among individuals, other than this being a relatively flexible distribution for continuous non-negative values?

***Re:*** *The gamma distribution is very common choice when modelling individual heterogeneity for a parameter. We have added a comment on this in the section of “Materials and methods” (Page 3 line 77--78).*

- Minor point (line 94): I prefer to reserve the term “genealogy” for describing the ancestral relationships among individuals in a population, and “phylogeny” for the ancestral relationships among populations. Thus the variable G represents a phylogeny relating infections sampled from different hosts. However, I also recognize that others refer to this tree as a “genealogy” or use these terms interchangeably.

***Re:*** *We used the term of “phylogeny” to represent the ancestral relationships among populations where the branch length is in genetic distance. We used the term of “genealogy” to represent the time-scale phylogeny where the branch length is in calendar time (Page 4, line 112--113).*

- line 102: “… or in relative terms as the coefficient of variation CV_\lambda = \frac{\sigma_\lambda}{\mu_\lambda}.” For readers who are less familiar with the CV, it would be helpful to note that this is a dimensionless quantity where standard deviation, as a measure of dispersion, is normalized by the mean that facilitates comparisons across data sets.

***Re:*** *Thanks for your suggestion! We changed the description of CV (Page 4 line 121--122).*

- lines 165-174: This section would greatly benefit from a diagram explaining the relationships among d_i, e_i, l_b, etc., as there is a substantial amount of notation being introduced here.

***Re:*** *Thanks for the suggestion! We revised fig 2 and added an example to illustrate these concepts (page 22).*

- line 369: Please provide methodological details on the BEAST analysis, i.e., model assumptions, prior distributions, assessment of convergence.

***Re:*** *We briefly described the implementation of BEAST in section “Real epidemiological data and genealogical reconstruction”in the supplementary file (Page 4 of the supplementary file).*

- Text S4: “During this simulation, when there is a newly diagnosed individual, the corresponding NCE value is calculated and the decision of contact tracing has been made based on the NCE value.” Is there a delay in this simulation for the time between diagnosis and sampling (sequencing), which would be required to compute the NCE value associated with that individual?

***Re:*** *As we assumed, there is no delay between the times of diagnosis and sampling (Page 9, line 315).*

- Figure 1: The notation in the figure does not correspond to the figure legend. For example, “the i-th individual was infected at t_i” – neither symbol appears in any of the figures. It seems that the authors switch between Roman numeral notation for branches and Arabic numerals for the time axis.

***Re:*** *We thank the referee for pointing this out. We revised the description of Figure 1 (page 21).*

- Figure 2. “The internal branches with the same label represent the infectious period of a particular individual.” But what if the start of the infectious period does not coincide with the branching point in the virus genealogy? This statement seems to assume that there is no within-host variation and that individuals are infectious immediately following transmission.

***Re:*** *Yes, we assumed no within-host variation and that the transmission event coincides with the branching event in the genealogy (Page 4, line 109-111). Our algorithm was developed under these assumptions, and we test the robustness of the algorithm by releasing these assumptions in simulation.*

- Figure 4. The data series for D=100 and \rho_{SD} = 1.0 should be identical between plots A and B, but they do not seem to be.

***Re:*** *Thanks for pointing this out. We performed simulation again and revised the figure (Page 24).*

**Reviewer #2:** With this manuscript the authors propose a method to estimate the amount of transmission heterogeneity based on virus sequence data in a sampled epidemic and suggest useful applications for the method. While the method is very interesting and the implementation of heterogeneity is simple but novel, the range of simulated situations is rather limited, e.g. it was only tested for a single R0 value (2.5). Hence, I suggest to ensure the method is indeed applicable to a wide range of situations.


Further comments and more detailed suggestions for simulation scenarios:
 
 
ll 115-117: how does that compare to the model presented here?

***Re:*** *Thanks for this suggestion! We cited some related works in the “Introduction” (Page 2 line 19--34) and made a comparison on simulated data with one of these method in the Result-section (Page 10 line 364--384).*

l 260: please specify how the R0=2.5 is derived from the estimates in [22] (which was 2.29 and 2.92, with and without rate heterogeneity among subepidemics resp).

***Re:*** *R0 = 2.5 is close to the mean of 2.29 and 2.92, so we chose 2.5 to show the performance of our algorithm. We also changed the recovery rate of the infected individuals to show the performance of our method under a variety of situations in the section of “Result” (Page 10 line 352--363).*

ll 282ff & Fig 3: the upward vs downward bias changes at CV~SQRT(2) for the 3 parameters R0, mu, gamma. and the same is seen in Fig 5. I doubt this is a coincidence, but that it has to do with the characteristics of the gamma function employed to model heterogeneity (if I understand correctly CV_lambda is the inverse squareroot of the shape parameter, is that right?). the authors should explore this and discuss how this may be used to assess in which direction they expect bias to occur.

***Re:*** *Thanks for pointing this out! We provided a detailed explanation about the biases in these estimates in the section of “Result” (Page 9 line 332--350)*

simulations:
- what if sampling is delayed?  - this would probably make the inference harder but would be more realistic. I suggest to add a simulation scenario to test that.

***Re:*** *In our model, the sample is not assumed to be taken at the time of transmission, but after a period of exponentially distributed length. This period is referred to as “infectious period”, and its length is drawn from an exponential distribution with the same rate at the time of infection for each infected individual (Page 3 line 83 - 86).*

real data analyses (page 10 / Fig 7): 
- please provide more information on the real datasets, at least the sampling period and number of samples
- please perform additional simulations that test significance of the real dataset analyses, i.e. (i) a slow outbreak with high heterogeneity and (ii) a fast outbreak with low heterogeneity, and compare the results to each other and the real data analyses resp. 
- why was a coalescent skyline used in the BEAST analyses? the tree prior may impact the tree characteristics esp. in the MSM_B dataset, which appears quite small, such that the coalescent skyline may be "overfitted"
- I would guess that the low gamma estimate in IDU_B is an artefact of the root of the tree ranging much further back than the oldest sample. this translates to a very long infectious period for the first cases, but is more likely a bias in the sampling process. can the model account for that by letting the sampling process start later?

***Re:*** *We thank the reviewer for these detailed suggestions. We have added the number of taxa and sampling time intervals to the data descriptions (Page 13 line 508--519).*

*Note that these data have been previously published and described in detail elsewhere. The use of these datasets here are to illustrate the application of our methods to real data, where we know from epidemiological data what happened. We used the skyline method because it was used in the original publications that described these data. It is the most popular model for this type of data because it makes the least assumptions about the population growth in the data. The long distance to the root in the IDU_B data may also be the result from incomplete sampling. There are many reasons for why it will be very common to have phylogenies that go further back in time than the sampling, e.g. there may have been original donors that no longer live in the sampled region, or there may be more than one importation to a region making a sample of the original donors impossible.*


phylogeny guided contact tracing:
- how robust is the NCE to biased sampling processes? parts of the tree that are sampled more heavily will have higher NCEs.
- this needs to be tested using simulations - applying heterogenous sampling schemes
- with large enough datasets a more complex birth-death model may be used to infer such differences, which could then be used to adjust the NCE accordingly.

***Re:*** *We agree that biased high sampling would be detected as compared to less sampled, but equally infectious parts of a tree. Thus, a method would have to be developed to detect the difference in NCE stemming from biased sampling versus truly elevated number of cases. However, biased, more dense sampling, is only possible if there truly were more infections, and would thus not miss truly active parts of a tree. Biased to less dense, on the other hand, may lead to missed transmissions at higher NCE values. While this may be addressed by further development of an alternative BD model as the reviewer suggests, this is not a trivial undertaking and we believe this is best left for future development.*

ll 452-453 "the effect of within host diversity is to make short branches only slightly longer whereas longer branches are increased more due to within host diversity"  - why is that?

***Re:*** *Diversity increases (linearly) after infection; thus, after a short time there is less diversity, leading to a small pre-transmission interval, while after longer time diversity is large, which may cause a larger pre-transmission interval. Therefore, a donor that has been infected for a longer time may transmit a lineage that has a larger pre-transmission interval than a donor who was more recently infected themselves.*

Fig 8 & ll 491ff are 20% and 50% realistic proportions for contact tracing? please provide some references for that

***Re:*** *Here we performed the simulation study under the scenario of Cross-sectional sampling. Under this scenario, we select the individuals for contact tracing only at the beginning of the prevention period (denoted as t_I_). We select a fraction (i.e.,20% or 50% ) of sampled individuals before time t_I_ for contact tracing either randomly (random prevention), or based on the descending order of NCE value (NCE based prevention), or based on the descending order of times since diagnosis (MRD prevention). Please see the subsection of “Calibration of Prevention Strategies” in the supplementary file for detailed discussion (Page 5 – 6 in the supplementary file).*

-  Fig 8 would be easier to read if the authors used a colour gradient for the fraction contact traced

***Re:*** *Thanks for this suggestion! We revised the figure to make it easier to read (page 28).*

ll 509-510: this has only been tested for very specific cases. please phrase this specifically referring to what you've tested, it's an unjustified generalization.

***Re:*** *We check robustness with respect to lots of different deviations from our model, including the finite susceptible population size, the biased sampling fraction, the change of time scale, and the variation of transmissibility with time since infection. We do this one at a time keeping other parameter values at common values. To vary the other parameters simultaneous would results in too many different comparisons. We have now made that clearer in the Discussion section (Page16 Line 663-681).*

Discussion: there may also be heterogeneity through time, not only among individuals, particularly regarding the time to diagnosis

***Re:*** *Thanks for these suggestions! We added some simulation results under the situation with heterogeneity through time in the section of “Result”(Page 11, line 409--440). As to the heterogeneity in the time to diagnosis, we agree that diagnoses occur at different times after infection for different persons. We have now made that clearer and discuss this in the Discussion (Page17 line 701-707).*

Parts of the supplement are confusing, e.g. the last sentence on page 3, most of Section 4.2 etc.

***Re:*** *Thanks for this suggestion. We revised the section 4.2 in the supplementary file to make it easier to read (Page 3 and 5 of the supplementary file).*

**Reviewer #3:** This manuscript introduces a new method to estimate transmission heterogeneity from viral sequence data. It is an important question and a successful such method is appropriate for publication in PLOS CB.

I have some concerns about the method and its assumptions.

Line 72 I don’t understand why you can say that the ratio of sequenced diagnosed over all infected (c /(a+b+c)) is the sampling ratio (c/(b+c)) unless a=0. Please rephrase, as this obviously isn’t what you mean.

***Re:*** *Thanks. We revised the description. The sampling ratio is c /(a+b+c) while the sequencing ratio is c/(b+c)(Page 4 line 92--96). The former is usually unknown while the latter is probably known in the analysis of data.*

Line 86 as you later clarify, you still can’t say G=T even when there is no in-host diversity, because T needs information about which host internal nodes are in (ie direction of transmission).

***Re:*** *T is referred to the transmission tree without information about who-infected-whom (Page 4 line 101). So we can roughly say that* G=T *if there is no within-host diversity (Page 4 line 104-105).*

A birth-death model with constant rates (even rates that vary between individuals) is a very poor match for the within-host natural history of HIV infection, which has an early high-transmission phase, followed by a long chronic infection phase, and then followed by progression to AIDS (in the absence of treatment). With treatment, the assumption that transmission ceases is good in some settings (with great treatment adherence and little resistance, for example), and poorer in others. But in pretty much all settings, assuming a constant infectivity for 2.5 years is not a great model; this needs to be discussed.
The simulation study could explore model mis-specification in this direction.

***Re:*** *Thanks for these suggestions! We added some simulation results under the situation with heterogeneity through time in the “Result” section (Page 11 line 409--440).*

Line 140 surely the likelihood is proportional to, not equal to, as k and theta have moved across the conditioning.
Does * mean multiplication in Eq (1), not convolution? –

***Re****: Yes,* ** means multiplication, and a proportionality symbol has now been inserted (Page 5 line 161).*

What is gamma^1_DG? - Ah, in line 143 you state that the indicator is for whether the infectious period ended before the observation period; surely the probability of this depends on when the infectious period started.
Pairwise transmission models have a long history and are well-liked in the community, but I have always wondered what the independence assumption means. Clearly if some individual A is in the dataset, then someone infected A (or A is the index case). If J infected A, then B did not infect A, nor C, D, …, because A can only have one infector. When we assume independence of all the transmission events, we cannot take these constraints into account.

***Re:*** *The independence assumption means that all the transmission events happen independently. In other words, the transmission events caused by individual A are independent of those caused by B. This independence assumption means we describe the process forward. And based on this assumption we can obtain the likelihood function. We revised the description in the manuscript to clarify these points (Page 5 line 154--157)*

Line 153 i don’t know what $(d_i, x_i)_{i=1}^n G$ is.

***Re****: Thanks! We corrected it.(Page 6 line 175)*

Line 163 - why would eq (1) be valid for a partially reconstructed transmission history? Or, what does that validity mean, given that the partially sampled history does not contain the information required to use (1) as it does not contain d_i and x_i (since it’s partially sampled)?

***Re:*** *The eq (1) is based on complete sampling. That is, we assumed that all the infected individuals are sampled (Page 5 line 150-152). Later this assumption will be relaxed in the analysis of sampled genealogy (started from Page 6 line 217).*

*Eq (1) is valid for a partially reconstructed transmission history because the transmission events caused by one infected individual are independent. Suppose that A infected B, C, and D at time t1, t2 and t3 respectively. We only observed the latter two transmission events at time t2 and t3, but we can still build the likelihood function based on these two events with eq (1) because these three transmission events are independent and that the composite likelihood is the product of three terms (Page 5 line 155-157).*

Line 183 - what does “each slice consists of branches having the same maximum number of branches to the tips” mean?

***Re:*** *In Fig 2 (page 22), we added an example to explain the definition of slice. Also we gave the definition of “slice” in Page 6 line 189-191.*

I like the observation in line 186 that the sum of the infectious periods has to add up to the total branch length; this applies to other models relating transmission to inference (phybreak, beastlier etc also) as far as I can tell.

***Re:*** *Thanks!*

Please state exactly what the CA algorithm does - alternating between relabelling and re-estimating sounds good but please give more details; pseudo-code.

***Re:*** *Thanks for your suggestion. We added a pseudo-code of the CA algorithm in the page 7 of supplementary file .*

It’s the section on analysis of the sampled genealogy where I really don’t follow and begin to doubt the modeling. First - is $t$ counted from t=0 at the root of the tree, or back into the past from the tips (and if so, which one)?

***Re:*** *$t$ counted from t=0 at the root of the tree (Page 4 line 115).*

With no in-host diversity, are you assuming that the external branches represent time from infection to sampling?

***Re:*** *No. The external branches represent time from the last transmission event to sampling.*

Why use L_p in the way that you do? Or do you mean the heights of the tips, in line 196?

***Re:*** *Since the sampled genealogy suffers from partial sampling, we were trying to find out a local part of the genealogy to which the CA algorithm apply because the CA algorithm was developed under the assumption of complete sampling.*

*Because of the constant diagnosis rate, the individuals infected earlier are more likely to be sampled at the end of observation, while those infected recently are more likely to be unsampled. Based on this consideration, the earlier part of genealogy (i.e., the part closer to the root) is more likely to be complete sampling. In other words, there is few individuals unsampled at the end of the observation. So we applied the CA algorithm to the earlier part of the genealogy (up to time* t – Lp*, where t is the time of stopping observation).* L_p *means how far we go back in time.*

*We revised the description in line 231-235 (page 7) to clarify the usage of L_p.*

You split the tree into the part before time t-Lp. There you estimate the sampling ratio; then you apply the CA algorithm.; then you correct the parameter estimates for Lp, and then “the results under different values of p are averaged” -- again, please clarify; give pseudo-code if possible.

***Re****: Thanks for your suggestion. We added pseudo-code in page 8 of the supplementary file.*

I really don’t see why the sampling ratio of the G(s) (t-Lp) is rho_SD *p . You have *set* p, so p could be whatever you choose. How does Lp provide an estimation about the p percentile for the distribution of infectious periods, and how does this mean that you know the sampling ratio of G(s)(t-Lp)?
Also, if the sequencing ratio (and sampling fraction) is fixed over time, then you know from the times of sequenced cases quite a lot about the system.

***Re:*** *As we defined $L_p$ it is the $p\times 100$ quantile of the lengths of the external branches of $\mathcal{G}^{(S)}$, so $L_p$ provides a conservative estimate of the $p \times 100$ percentile for the distribution of infectious periods. Hence, we assume that a fraction $p$ of the individuals infected within [0, $t-L_p$] will be diagnosed before time $t$.*

*In addition, since the sequencing ratio is rho_SD, the composite sampling ratio of the $\mathcal{G}^{(S)}$ is rho_SD *p.*

*We revised the description in the manuscript to make it easier to read (Page 7 line 231--240)*

Line 220 what is “upscaled” here?

***Re:*** *please see the response to the last question.*

Line 244 why should taxa with higher NCE values be more likely to be phylogenetically linked to unsampled highly infectious individuals? Does this happen in your simulations? I might have thought that in densely-branching parts of the phylogeny, sampling is likely to be more dense, not less.

***Re:*** *Sampling is assumed to be random. Thus, a more dense part of a tree (higher NCE) indicates more transmissions. Because super-spreaders (or outbreaks) have more transmission contacts in a time period, they will be more likely in dense parts of a tree. It is possible that one of the sampled individuals is the super-spreader, but it is also possible that the super-spreader has not been sampled. However, concentrating contact tracing to the individuals in dense parts will more efficiently find the super-spreader and reduce the epidemic as we show in our simulations.*


R0=2.5 seems a high estimate for HIV in Switzerland; most estimates for developed countries without generalised epidemics are less than 1.

***Re:*** *In ref [22] (Estimating the basic reproductive number from viral sequence data. Molecular biology and evolution. 2011;29(1):347–357.), the estimated R0 was 2.29 and 2.92, with and without rate heterogeneity among subepidemics respectively. So we added more simulation under the condition of R0 = 1.5, 2, 2.5, and 3 to show the performance of our algorithm under a variety of situations (page 9 line 352--363).*

How robust is the method to model mis-specification? As above I would be most concerned about the non-constant transmission rate.

***Re:*** *Thanks for your suggestion. We added some simulation results under the condition with heterogeneity through time in the manuscript (page 11 line 409 -- 440).*

I think the main text should state more about the simulation method, as it is inconvenient to have to go and dig it out of a supplement; in particular, the fact that you simulate trees rather than sequences is quite important as it means that you cannot see how robust the method is to tree uncertainty; given that you required timed trees, there is also the potential for the nuances of timed tree reconstruction to contribute to bias or noise in the estimates.
Similarly, the main text should have information about how the within-host diversity was simulated (perhaps with a figure of a tree from such a simulation).

***Re:*** *We have clarified in the main text that we simulate time-scaled trees and not actual HIV sequence populations (page 11 line 444-447). The uncertainty in the reconstructing and timing of the trees will certainly affect our method, but this is beyond the main scope of our study. As to the simulation of within-host diversity, we used an existing method published elsewhere to add within-host diversity to the genealogy. The simulation study with within-host diversity is to show the performance of our method under this realistic situation. So the details of simulation of within-host diversity is beyond the scope of our study.*

There are various plots with “coefficient of variation” as the title and throughout there are plot titles but no y axis labels, which left me confused. Fig 6 - CV_lambda is on the x axis; what is y. I can’t tell from the caption what the vertical axis is showing. In Fig 3, CV_\lambda is defined as “levels of heterogeneity” - what is it, in terms of the parameters of the simulation model?

***Re:*** *CV_lambda is the coefficient of variation, and is a dimensionless quantity where standard deviation is normalized by the mean, i.e., $CV_{\lambda}=\sigma_{\lambda} /\mu_{\lambda}$. We defined CV in the manuscript (page 4 line 121) and also revised the legend of Fig 3 (page 23) to make it easier to understand.*

Figs 9, 10 what is on the y axis? The title “comparison of relative effect” doesn’t tell us much

***Re:*** *Thanks for your suggestion. We added the y axis labels for Fig 5,6,9, and 10 (page 25,26,29, and 30) to make them easier to read.*

Supplement S2
Why is the adjusted transmissibility just lambda_i/rho ?

***Re:*** *This question is related with the previous question of “Line 220 what is “upscaled” here?”.*

*In our model, the transmission rate lambda_i was defined as the number of secondary cases caused by i-th individual during a unit of time. In the analysis of sampled genealogy, we firstly applied the CA algorithm to the local part of genealogy by assuming there was no unsampled individuals, resulting in the estimated lambda_i. Later, in the correction, we allowed for the unsampled individual based on the estimated sampling ratio rho. So the estimated transmission rates of the sampled individuals shall be upscaled equally as lambda_i/rho (page 8 line 262-265). We also revised the section of S2 (page 3) in the supplementary file to make it easier to read.*
