# Supplementary material for: Inferring transmission heterogeneity using virus genealogies: Estimation and targeted prevention
Source: PLoS Comput Biol. 2020 Sep 3;16(9):e1008122. doi: 10.1371/journal.pcbi.1008122 (PMC7494101; doi:10.1371/journal.pcbi.1008122)
Supplement: Attachment — Submitted filename: re2reviewers.docx [file pcbi.1008122.s012.docx]

*Introduction, lines 24-34: The authors’ characterization of the multi-state birth-death (MSBD) model is incorrect. The MSBD model does not require the positions and times of state changes in the phylogeny to be known in advance — instead, the optimization algorithm adds and removes state changes in the phylogeny.*

**Response**

Thanks for pointing this out! We revised the description about the MSBD and related methods (line 27-34 page 2)

*Results, lines 364-384: Your comparison of the uncorrelated model to the MMPP model is problematic because you have simulated transmission rates as being drawn individually (in an uncorrelated fashion) from a gamma distribution. This violates a central assumption of the MMPP, which is premised on the existence of correlated rates (which is how clusters are identified). It is therefore not at all surprising that your model performs well on data simulated under the same model, and MMPP performs poorly when its core assumption is broken. The honest approach would be to evaluate the methods under both uncorrelated and correlated scenarios, recognizing that MMPP should be unable to detect variation in transmission rates in the uncorrelated scenario. In addition, MMPP does not attempt to estimate R0 — it is misleading to present results (Figure S9) that indicate otherwise.*

**Response**

Thanks for this suggestion! We added more simulation studies under the situation with autocorrelation of transmission rates, changed the way of presenting the simulaiton results, and revised the description about the comparison of these two methods (Page 10 line 368-412 and Figure S9 in supplementary file).

*Introduction, lines 45-52: The addition of this text helps clarify that the authors’ model assumes that variation in transmission rates are uncorrelated (independent draws from some parametric distribution). However, the rationale provided by the authors — “varying degrees of social activity” — implies auto-correlation in transmission rates, since risk behaviours exhibit a high degree of homophily. Varying viral loads is time-heterogeneous within individuals, whereas your model assumes rates are time-invariant at the level of individuals. Thus, the rationale for your model remains unclear.*

**Response**

Thanks for this suggestion! It is true that our model was developed under a relatively simple situation by assuming no autocorrelation of transmission rates, but we evaluated how our method performed in the presence of autocorrelation with simulation datasets. We added these explanations in the manuscript (Page 3 line 64-66).

*Methods, lines 261-280: The addition of this section helps clarify the core assumptions of the authors’ model. Another way to put this is that the authors assume that, in the setting of high variation in transmission rates, all “superspreader” individuals have been sampled and diagnosed. The authors need to reconcile this assumption with another central assumption of their model, that the sampling and diagnosis of cases from the epidemic is occurring at an early exponential phase of the epidemic. (This would involve a tremendous sampling effort because most individuals will be uninfected when sampled.)*

**Response**

Thanks for pointing this out! In fact, we did not assume that all “super-spreading” individuals have been sampled, but assumed that most of the coalescent events in the sampled genealogy were corresponding to the transmission events caused by these “super-spreading” individuals. Note that the transmission event caused by an individual could be reconstructed by the information from its descendants. For example, individual A infected B and then infected C. When A was unsampled while B and C were sampled, the coalescent event of B and C in the sampled tree (with tips B and C) is coincide with that of A and B in the full tree (with tips A~C). A “super-spreading” individual has more descendants than a normal individual, so the tips in the sampled tree are more likely to be descendants of the super-spreader. Hence the coalescent events in the sampled tree are more likely caused by the super-spreader even if it is not sampled. We added these explanations in the manuscript (Page 8 line 270-273).

*For equation (9), I can see that the authors intend to scale the constant transmission rate for unsampled individuals from \mu_lambda^0 at CV=0 to \gamma as CV\leftarrow\infty. I cannot reconcile this with the statement at the top of this paragraph that \lambda _{us} = \mu_lambda^0 / \rho. It is also important to clarify for the reader that there is no variation in transmission rates among unsampled individuals under any level of CV in this model.*

**Response**

Thanks for pointing out this! There was a typo, that is, under the homogeneous situation \lambda _{us} = \mu_lambda^0. I corrected it in the manuscript (page 8 line 266).
